# Expression of Toll-Like Receptors on Human Rectal Adenocarcinoma Cells

**DOI:** 10.1007/s00005-013-0260-z

**Published:** 2014-01-05

**Authors:** Marcin Tchórzewski, Przemysław Lewkowicz, Adam Dziki, Henryk Tchórzewski

**Affiliations:** 1 Department of General and Colorectal Surgery, Medical University of Lodz, Lodz, Poland; 2 APC Medical Analyses, Lodz, Poland; 3 Department of Neurology, Laboratory of Neuroimmunology, Medical University of Lodz, Lodz, Poland

**Keywords:** Colon cancer, Cell culture, TLR2, TLR4 expression

## Abstract

The innate immune system uses Toll-like receptors (TLR) to detect the presence of pathogen patterns thus allowing for rapid host defense responses. Stimulation of TLR results in inflammatory response and regulatory cytokine production affecting acquired immunity. The aim of the study was an evaluation of TLR2 and TLR4 expression on the surface of human colon cancer cells in primary culture with or without autologous peripheral blood mononuclear cells. Surgical specimens of colon cancer were processed to obtain cancer cells. Cancer cells separation was conducted first by mechanical tissue disintegration and than by gradient centrifugation to obtain 95 % cell confluence. By staining the isolated cells the pathologist determined them as adenocarcinoma. Colon cancer cells were then co-cultured in 24 h culture alone or together with autologous lymphocytes. Reverse-transcription polymerase chain reaction was performed for detection of TLR2 and TLR4 mRNA in colon cancer and normal colon epithelial cells using commercially available primers. Resting as well as phytohemagglutinin or lipopolysaccharide (LPS) stimulated cells were tested. Receptor proteins on cancer cells were examined by immunohistochemistry. TLR4 mRNA was detected in cancer cells. Autologous lymphocytes do not exert any effect on these receptors expression. TLR4 mRNA expression was not observed in normal colon epithelial cells. TLR2 mRNA was present on LPS stimulated cancer cells as well as on resting and stimulated lymphocytes. Expression of TLR2 and TLR4 receptor proteins on colon cancer cells were confirmed by immunohistochemistry. TLR4 may be responsible for uncontrolled tumor growth under LPS stimulation in human colon environment.

## Introduction

Toll-like receptors (TLRs) are a group of cell surface receptors recognizing specific structures on the surface of pathogens or the specific elements of their own proteins or tissues. The name was created, when receptor of the *Drosophila melanogaster* embryo was first described (Means et al. 2000; Takeda and Akira 2004). With the progress of research, the significant structural similarity of TLRs present in various species including mammals and humans was demonstrated. TLRs are innate immunity receptors recognizing the specific structure of the pathogen. Exogenous agonists including bacterial lipopolysaccharides (LPS) of Gram-negative bacteria, mycobacteria, yeast and endogenous retroviruses stimulate TLRs, similarly to other agonists such as hyaluronians, heparans, heat shock proteins (HSP60, HSP70), fibrinogen and surfactants regardless of species (Johnson et al. 2003). Previous immunization is not necessary to identify the pathogen, which is prerequisite in case of acquired immunity.

Experimental research but also clinical observations suggest that these receptors may play an important role in the pathogenesis of colon cancer. Population studies have shown that specific polymorphism of TLR2 and TLR4 receptors significantly increases the risk of colon cancer development in individuals already loaded with other known environmental factors (obesity, smoking, etc.) (Pimental-Nunes et al. 2013). They may be a predictor of increased cancer risk in people with colon hyperplastic polyps (Eiro et al. 2012). Increased expression of TLR4 in colon cancer (stroma) significantly accelerates the development of the disease and is a poor prognostic factor (Cammarota et al. 2010). This effect may be dependent on TLR4 stimulation by LPS present in the environment of the intestine. This was proved in studies on SW480 colon carcinoma cell line when LPS stimulation induced growth of cancer cell lines in vitro (Rakhesh et al. 2012).

Pathogenic role of tumor infiltrating lymphocytes T (CD3) is not understood completely. Patients with colon cancer, stable micro satellites and massive infiltration of T lymphocytes better respond to the treatment. Patients whose tumors are highly infiltrated by T cells have a beneficial prognosis compared to those with trace infiltration (Dahlin et al. 2011). The high density of CD45RO^+^ cells in metastases was also a good prognostic factor of survival in patients at stage IV colon cancer (Lee et al. 2013), while patients with small number of tumor infiltrating lymphocytes are at high risk of disease recurrence (Kocián et al. 2012).

The study was undertaken to evaluate the expression of TLR 2 and TLR4 mRNA in primary in vitro colon cancer cell culture (not cell line). Cells were isolated from tumors of patients undergoing potentially radical surgery for cancer. TLR expression and the effect of autologous non-stimulated and stimulated peripheral blood lymphocytes on the TLR expression was examined. The proposed study relates to some extent to the in vivo reactions where peripheral blood lymphocytes mimic lymphocytes that infiltrate the tumor.

## Materials and Methods

The study was performed using colon cancer cells from tumors of five patients who underwent surgery at the Department of General and Colorectal Surgery, Medical University of Lodz, after obtaining their written consent. Peripheral blood was collected on heparin several hours before surgery. Peripheral blood mononuclear cells (PBMC) with 96 % homogeneity as evaluated morphologically were isolated from peripheral blood using Gradisol (Aqua-Med, Łódź, Poland) gradient separation. The nondistributed whole population of PBMC was used to avoid separation procedures dependent on unspecific stimulation.

Colon cancer cells were isolated from tumor specimens taken intraoperatively 2–3 h after surgery. All tumors used in the experiments were of polypoid or infiltrating morphology. Ulcerative lesions were discarded because of bacteria contamination. The histopathological examination confirmed cancer cells as adenocarcinoma. In mucinous adenocarcinoma the most common types are columnar or goblet cells with component of endocrine cells, tumor infiltrating lymphocytes and neutrophils. Cancer elicits an inflammatory and desmoplastic reaction. The tumor may invade all layers of the bowel wall and pericolic fat, permeating perineural space and/or invade veins. All tumors used in the study were graded as T2N0M0 or T3N0M0 (T-primary tumor, N-regional lymph nodes, M-metastases).

Tumor tissue underwent delicate mechanical disintegration by pressing through the steel mesh, collected cell suspension was purified from tissue residues on microfilters (Difco, USA) and then centrifuged on Lymphoprep gradient to separate cancer cells and tumor infiltrating lymphocytes.

Stabilized smears of analyzed cancer cells suspensions were stained with hematoxylin and eosin. Microscopic picture revealed single or grouped glandular cells. The cells had large hyperchromatic nuclei and abnormal chromatin structure. Nuclei of some cells displayed prominent nucleoli. Cell cytoplasm was partially disorganized and whole picture corresponded to atypical adenocarcionoma cells with different degree of pleomorphism.

The specimen of an unaffected intestine was also taken from the distant healthy part of the same intestine. Similarly to the tumor, specimen of the normal intestine underwent analogous processing and served as a control group. Obtained cells were epithelial cells of the intestinal mucous.

Tumor cells represent cancers from which they were derived. The cell suspensions were evaluated morphologically by pathologist and 95 % of cells were confirmed as cancer cells. The cancer cells were then suspended in RPMI 10^6^/ml (Biomed, Lublin, Poland) supplemented with 10 % foetal calf serum (Difco, USA) and antibiotics and then cultured in 0.5-ml well plates for 24 h. Phytohemagglutinin (PHA; Difco, USA) 10 μg/ml, LPS (Difco, USA) 10 μg/ml or autologous lymphocytes (10^5^/ml) were added to the cancer cell cultures to overall concentration of 10^6^/ml (10:1 ratio). The culture system is shown in figure description. The cells were then washed with RPMI and the presence of mRNA for TLR2 and TLR4 were determined by reverse-transcription polymerase chain reaction using the commercially available primers for human TLR2 and TLR4 in accordance with the manufacturer’s instructions (Super Array, USA). The synthesis of complementary DNA was performed in commercial system (Supper Array, USA) using 5 μg of isolated mRNA.

The TLR2 and TLR4 receptors expression at the protein level were evaluated applying immunohistochemistry procedure. Cryostatic slides 5 μm thick were prepared in −30 °C on MTE cryostate (SLEE Technik, Germany). Cold acetone fixed sections (10 min in 14 °C) undergone heat-induced epitope retrieval procedure in antigen retrieved reagent-acidic 2 min in 92 °C (R&D system, USA) than the sections were washed in phosphate buffered saline (PBS; Wytwórnia Surowic i Szczepionek, Poland) and blocked with 10 % goat serum (Santa Cruz, USA) and 0.3 % Triton X-100 (Sigma Aldrich, USA) in PBS for 20 min in room temperature. The slides were incubated for 21 h with primary anti-TLR2 [5 μg/ml (Abcam, Cambridge, UK)] and anti-TLR4 antibodies (5 μg/ml; clon HTA 125 Enzo Life Sciences) in PBS supplemented with 0.3 % Triton X-100, 0.01 % sodium azide and 2 % goat serum. Than the secondary goat anti mouse IgG1 heavy chain fluoresceine preadsorbent antibodies 2.5 μg/ml were added (Abcam, Cambridge, UK). Cell nuclei were stained with 1.5 μg/ml of DAPI (4.6-diamidino-2-phenylindole) for 30 min at room temperature (UltraCruz Mounting Medium, Santa Cruz Biotechnology, USA). To evaluate the nonspecific binding the incubation with secondary antibodies was performed. The slides were evaluated in confocal microscope BD Pathway 855 Bioimager system equipped with Olympus 20/340 UAPO × 0.75 objective and IPLab Imaging Software (Rockville, MD, USA) which allows to quantify the complete fluorescence and mean fluorescence intensity. Unspecific signals were excluded with electronic fluorescence autosegmentation system.

## Results and Discussion

Most studies on the role of TLRs in carcinogenesis was performed on stable cell lines derived from colon cancer cells (Cammarota et al. 2010; Rakhesh et al. 2012). Mice experimental studies help us understand the relationship between genetic predisposition, infection-induced inflammation, neutrophils influx and the development of cancer (Boulard et al. 2012). Understanding the excitation of signaling pathways and the factors regulating cell functioning in the intestinal wall and the environment responsible for these receptor reactions is crucial. LPS present in the intestine is an agonist of TLR4 on colon cancer cell line, increasing the resistance to apoptosis but not affecting the expression of TLR4 (O’Leary et al. 2012; Slatter et al. 2012).

**Fig. 1:**
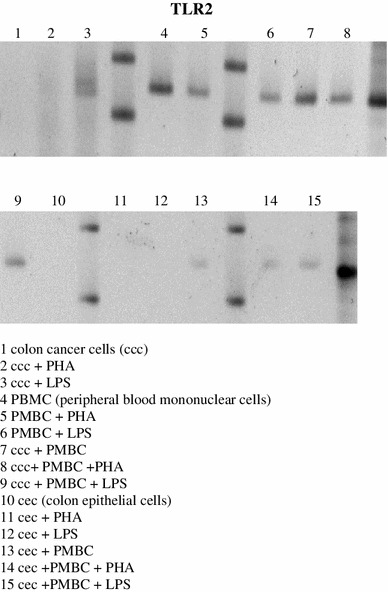
TLR2 mRNA expression was observed on LPS stimulated colon cancer cells in primary culture in vitro. Autologous PBMC expressed mRNA for TLR2, primary mixed culture with colon cancer cells does not affect above mRNA for TLR2 expression. Colon epithelial cells do not express mRNA for TLR2. The presented results are representative of five independent experiments

In our study, mRNA for TLR2 on cancer cells was observed after LPS stimulation. The presence of TLR2 mRNA on resting or PHA or LPS stimulated lymphocytes is clear. TLR2 mRNA expression in mixed primary cultures was observed in the presence of both non-stimulated and stimulated autologous lymphocytes (Fig. 1).

**Fig. 2:**
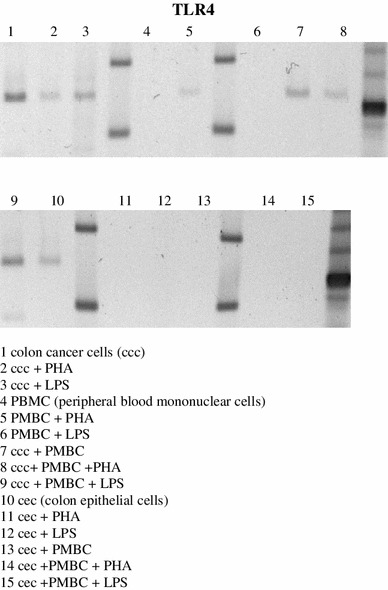
Expression of mRNA for TLR4 is present on colon cancer cells both resting and stimulated with PHA or LPS in 24 h primary culture in vitro. Both resting and stimulated autologous PBMC do not express mRNA for TLR4. Mixed culture of PBMC with colon cancer cells has no effect on intensity of mRNA for TLR4 expression. The presented results are representative of five independent experiments

The examination of colon cancer cells after 24 h of in vitro primary culture confirmed the presence of mRNA for TLR4 receptor on them. Stimulation with LPS did not affect the expression of the TLR4. There was no evidence of the presence of mRNA for TLR4 on resting or PHA or LPS stimulated autologous lymphocytes. Mixed cultures of tumor cells with resting, PHA or LPS stimulated autologous lymphocytes did not alter expression of mRNA of TLR4 on colon cancer cells. There was no expression of the TLR2 and TLR4 mRNA on normal or stimulated intestinal epithelial cells of the same patient as they were not visible in the mixed culture with autologous lymphocytes (Fig. 2).

**Fig. 3:**
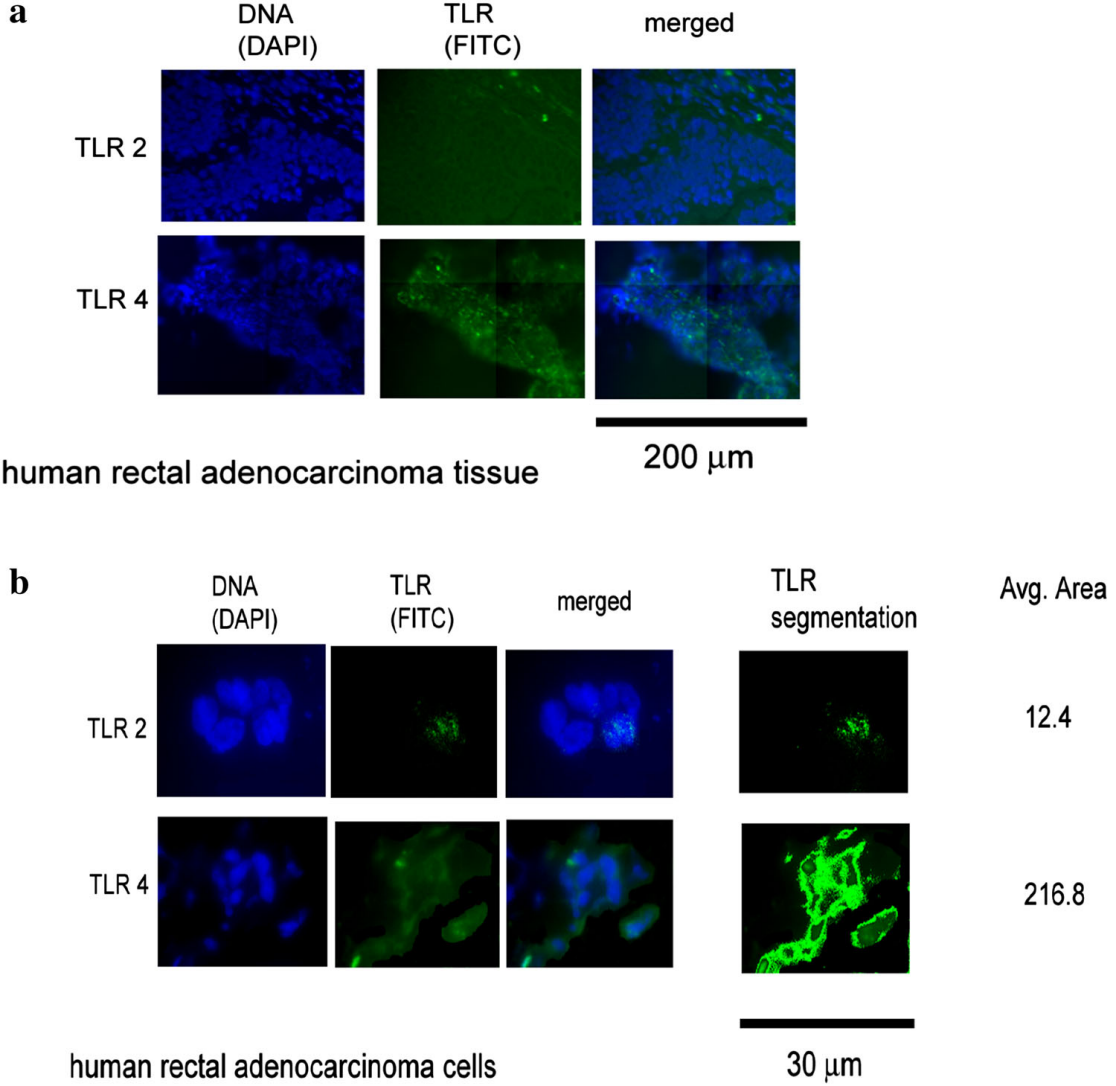
**a** Fluorescence signals for TLR2 and for TLR4 receptor proteins are visible in whole cancer tissue sections. **b** Fluorescence signal for TLR4 covers almost entire surface of the tumor cells, while the TLR2 is expressed as a point fluorescence only

Immunohistochemical studies confirmed presence of both receptors at the protein level on the tumor tissue sections and on single cancer cells. Mean fluorescence intensity of TLR4 was more intense than TLR2 (Fig. 3a, b).

The presented results of the expression of TLR2 and TLR4 mRNA on cultured colon cancer cells in 24 h primary cultures, give more reliable information because the cancer cells were collected directly from surgical patients, and receptor expression was examined under strict conditions after activation with LPS and in mixed cultures with autologous lymphocytes. Such setup to some extent mimics in vivo conditions and gives a view on tumors infiltrating lymphocytes function. Results suggest that own lymphocytes in these conditions did not affect the mRNA expression of TLR4, but may increase the expression of mRNA for TLR2. This may be of particular clinical importance. Studies performed on large groups of patients (Slatter et al. 2012) confirmed the relationship between TLR expression in the tumor environment and lifestyle and disease. It has long been suggested that the inflammation is a powerful stimulus for the development of colon cancer and facilitates the formation of metastases and relapses. LPS acting on TLR4 activates the formation of reactive oxygen intermediates in the colon cancer cell lines and these are the internal factors increasing the adhesion of endogenous cell to collagen and therefore increase the possibility of developing metastases (O’Leary et al. 2012).

In the experimental conditions a potent stimulator of SW620 colon cancer cell line is LPS-specific agonist of TLR4, recognizing LPS structure which is a reproduction of the pathogen (the called PAMPs: pathogen associated molecular pattern). The potency of LPS depends on the cylindrical or conical architecture of the lipid A (Netea et al. 2002), hence its different properties dependent on the LPS-producing bacterial strain. TLR4 stimulation by LPS results in the formation of immunosuppressive agents, increasing the resistance to apoptosis and inhibition of the immune response (Tang et al. 2010; Tang and Zhu 2012). Inhibition of TLR signaling may help for the prevention of cancer induction (Rakhesh et al. 2012). In summary, it can be stated that the expression of TLR4 on primary culture of colon cancer cells is the original contribution which points out the possible role of TLR4 in tumor survival and growth after local LPS stimulation.

## References

[CR1] Boulard O, Kirchberger S, Royston DJ (2012). Identification of a genetic locus controlling bacteria-driven colitis and associated cancer through effects on innate inflammation. J Exp Med.

[CR2] Cammarota R, Bertolini V, Pennesi G (2010). The tumor microenvironment of colorectal cancer: stromal TLR-4 expression as a potential prognostic marker. J Transl Med.

[CR3] Dahlin AM, Henriksson ML, Van Guelpen B (2011). Colorectal cancer prognosis depends on T-cell infiltration and molecular characteristics of the tumor. Mod Pathol.

[CR4] Eiro N, Gonzalez L, Gonzalez LO (2012). Study of the expression of Toll-like receptors in different histological types of colorectal polyps and their relationship with colorectal cancer. J Clin Immunol.

[CR5] Johnson GB, Brunn GJ, Tang AH (2003). Evolutionary clues to the functions of the Toll-like family as surveillance receptors. Trends Immunol.

[CR6] Kocián P, Sedicová M, Drgáć J (2012). K-ra mutational status and tumor-infiltrating lymphocytes in human colon cancer: state of the art and future perspectives. Rozhl Chir.

[CR7] Lee WS, Kang M, Back JH (2013). Clinical impact of tumor-infiltrating lymphocytes for survival in curatively resected stage IV colon cancer with isolated liver or lung metastasis. Ann Surg Oncol.

[CR8] Means TK, Golenbock DT, Fenton MJ (2000). Structure and function of Toll-like receptor proteins. Life Sci.

[CR9] Netea MG, van Deuren M, Kullber BJ (2002). Does the shape of lipid A determine the interaction of LPS with Toll-like receptors?. Trends Immunol.

[CR10] O’Leary DP, Bhatt L, Woolley JE (2012). TLR-4 signalling accelerated colon cancer cell adhesion via NF-κB mediated transcriptional up-regulation of Nox-1. PLoS ONE.

[CR11] Pimental-Nunes P, Teixeira AL, Preira C (2013). Functional polymorphisms of Toll-like receptors 2 and 4 alter the risk for colorectal carcinoma in Europeans. Dig Liver Dis.

[CR12] Rakhesh M, Cate M, Vijav R (2012). A TLR-interacting peptide inhibits lipolysaccharide-stimulated inflammatory responses, migration and invasion of colon SW480 cells. Oncoimmunology.

[CR13] Slatter ML, Herrick JS, Bondurant KL (2012). Toll-like receptor genes and their association with colon and rectal cancer development and prognosis. Int J Cancer.

[CR14] Takeda K, Akira S (2004). TLR signaling pathways. Semin Immunol.

[CR15] Tang X, Zhu Y (2012). TLR4 signaling promotes immune escape of human colon cancer cells by inducing immunosuppressive cytokine and apoptosis resistance. Oncol Res.

[CR16] Tang XY, Zhu YQ, Wei B (2010). Expression and functional research of TLR4 in human colon carcinoma. Am J Med Sci.

